# Watching sports and depressive symptoms among older adults: a cross-sectional study from the JAGES 2019 survey

**DOI:** 10.1038/s41598-021-89994-8

**Published:** 2021-05-19

**Authors:** Taishi Tsuji, Satoru Kanamori, Ryota Watanabe, Meiko Yokoyama, Yasuhiro Miyaguni, Masashige Saito, Katsunori Kondo

**Affiliations:** 1 Faculty of Health and Sport Sciences, University of Tsukuba, 3-29-1 Otsuka, Bunkyo City, Tokyo 112-0012 Japan; 2 Center for Preventive Medical Sciences, Chiba University, 1-33 Yayoi-cho, Inage Ward, Chiba City, Chiba 263-8522 Japan; 3 Teikyo University Graduate School of Public Health, 2-11-1 Kaga, Itabashi City, Tokyo 173-8605 Japan; 4 Department of Preventive Medicine and Public Health, Tokyo Medical University, 6-1-1 Shinjuku, Shinjuku City, Tokyo 160-8402 Japan; 5 Department of Rehabilitation, Tsushima City Hospital, 3-73 Tachibana-cho, Tsushima City, Aichi 496-8537 Japan; 6 Graduate School of Medical and Pharmaceutical Sciences, Chiba University, 1-8-1 Inohana, Chuo Ward, Chiba City, Chiba 260-8670 Japan; 7 Center for Gerontology and Social Science, National Center for Geriatrics and Gerontology, 7-430 Morioka-cho, Obu City, Aichi 474-8511 Japan; 8 Department of Social Welfare, Nihon Fukushi University, Okuda, Mihama-cho, Chita-gun, Aichi 470-3295 Japan; 9 Center for Well-Being and Society, Nihon Fukushi University, 5-22-35 Chiyoda, Naka Ward, Nagoya City, Aichi 460-0012 Japan

**Keywords:** Depression, Geriatrics, Epidemiology, Preventive medicine

## Abstract

The current study investigated the relationship between the frequency of watching sports and depressive symptoms among older adults. This study used cross-sectional data from the Japan Gerontological Evaluation Study, a nationwide mail survey of 21,317 older adults. Depressive symptoms were defined as a Geriatric Depression Scale score of ≥ 5. Participants were queried regarding the average frequency at which they watched sports on-site and via TV/Internet over the past year. Among the 21,317 participants, 4559 (21.4%) had depressive symptoms, while 4808 (22.6%) and 16,576 (77.8%) watched sports on-site and via TV/Internet at least once a year, respectively. Older adults who watched sports on-site a few times/year (prevalence ratio, 0.70; 95% confidence interval, 0.65–0.74) or 1–3 times/month (0.66, 0.53–0.82) were less likely to have depressive symptoms compared to non-spectators after adjusting for frequency of playing sports, exercise activities, and other potential confounders. Meanwhile, a dose–response relationship was confirmed for watching via TV/Internet (prevalence ratio of 0.86, 0.79, and 0.71 for a few times/year, 1–3 times/month, and ≥ 1 time/week, respectively). This study suggested that watching sports on-site or via TV/Internet, regardless of whether they regularly engage in sports, may reduce the risk of depressive symptoms among older adults.

## Introduction

To facilitate healthy aging among older adults, many studies in the fields of preventive medicine and public health have examined the relationship between sports/exercise activities and health outcomes, most of which have been in the context of “doing/playing” sports, exercise, or physical activity^1–3^. On the other hand, few reports have investigated the relationship between health status and “watching” sports^4,5^. The Second Sport Basic Plan established by the Ministry of Education, Culture, Sports, Science and Technology, which forms the basis of policy on sports promotion in Japan, encourages people to have various relationships with sports by not only playing but also watching and supporting sports^6^. In European countries, a continuing project called “Healthy Stadia” aims to promote community health by designating sports stadiums as a base for improving public health and by having various relationships with sports^7,8^. Watching sports is a positive activity that promotes excitement and enjoyment^9^ while also having an effect on socialization, such as fostering social cohesion in the community and strengthening social networks with friends or acquaintances. For instance, several studies in the field of sports management research have reported that attachment to teams and interaction with others were positively correlated with sports-watching behavior^10,11^.

Intervention studies on older adults have shown that subjective happiness increased after watching a professional baseball game^4^ and that depressive symptoms improved after 2 months of regularly watching baseball at a stadium^5^. Allowing older adults to attend a sports game on-site have also been found to increase their perceived emotional support from fans, leading to enhanced subjective wellbeing^5^. Despite the relatively small sample sizes (i.e., a few tens) and the pilot nature of the aforementioned studies, they showed that watching sports had a positive effect on the mental health of older adults. Furthermore, sports can be viewed not only on-site but also via TV or the Internet. In general, however, long screen times have often been reported to have adverse effect on health outcomes^12,13^. While a study focusing on watching sports TV programs among older adults suggested that it did not promote physical activity but rather increased the risk of obesity^14^, no study has investigated the relationship between watching sports and mental health.

Depression has remained a major issue in the mental health of older adults given its strong association with more functional and cognitive impairment than that in younger adults and with significant costs for the individual, family, and social security^15^. Therefore, the current study aimed to examine the relationship between the frequency of watching sports on-site and via TV/Internet and depressive symptoms using nationwide cross-sectional data for older adults in Japan. We hypothesized that fewer older adults who watched sports frequently would have depressive symptoms, regardless of whether they regularly played sports and engaged in exercise activities. Furthermore, social cohesion and networks could partly explain this relationship.

## Methods

### Study design

This study used cross-sectional data from the Japan Gerontological Evaluation Study (JAGES), an ongoing cohort study exploring social, environmental, and behavioral factors related to health deterioration, particularly functional decline or cognitive impairment, among individuals aged ≥ 65 years^16,17^. Between December 2019 and January 2020, a set of questionnaires were mailed to a total of 345,356 community-dwelling individuals aged ≥ 65 years selected from 60 municipalities, including metropolitan, urban and semi urban, and rural communities across 24 Japanese prefectures from as far north as Hokkaido (i.e., the northernmost prefecture) to as far south as Kyushu (i.e., the southernmost region). A random sample from official residents registered in the 43 large municipalities and a complete census of the older residents of the remaining 17 smaller municipalities were obtained. A total of 240,889 individuals responded to our mail, with a response rate of 69.8% (range, 54.4–89.8% throughout the 60 municipalities). A total of 29,444 participants responded to the question items on watching sports given that these were distributed to one-eighth of the participants who were randomly allocated. We excluded 8127 respondents who had no independence in activities of daily living, did not provide informed consent, or had no data regarding sex, age, or residence area. In total, data from 21,317 eligible respondents (10,324 men and 10,993 women) were used. Ethical approval for the study was obtained from the Ethics Committee of Chiba University, Japan (Approval number: 2493) and the National Center for Geriatrics and Gerontology, Japan (Approval number: 992–3). This study was performed in accordance with the principles of the Declaration of Helsinki. All participants were informed that participation in the study was voluntary and that completing the questionnaire, selecting the acceptance checkbox, and returning it via mail indicated their consent to participate in the study.

### Depressive symptoms

Depressive symptoms were assessed using the 15-item Geriatric Depression Scale (GDS)^18,19^. Following previous research^20,21^, mild or severe depressive symptoms (GDS ≥ 5) were set as the outcome of the present study. Cronbach’s α for internal consistency of the scale was 0.80, while the cutoff point was previously validated to have a 96% sensitivity and 95% specificity for screening major depressive disorder^21^.

### Frequency of watching sports

Partially modified with reference to the survey items of the Japan Sports Agency^22^, the following questions were used to determine the frequency at which participants watched sports: “How often have you watched sports directly at the site on average over the past year? These include not only professional sports, but also local sports clubs and groups” and “How often have you watched sports on TV or the Internet on average over the past year? These include not only professional sports, but also local sports clubs and groups (except for a little watching on TV news).” For each question, the following four responses could be provided: ≥ 1 time/week, 1–3 times/month, a few times/year, or zero.

### Covariates

While partially referring to factors that presumably confound the relationship between exercise activities and depressive symptoms among older adults^23^, potential confounders between watching sports and depressive symptoms were assessed. Participants were categorized into the following age groups: 65–69, 70–74, 75–79, 80–84, and ≥ 85 years. Given that the relationship between watching sports and depressive symptoms had no interaction according to sex, we did not perform sex-stratified analysis and treated sex as a covariate. The frequency at which participants played sports and engaged in exercise activities over the past year (zero, a few days/year, 1–3 days/month, 1 day/week, or ≥ 2 days/week), marital status (married or unmarried), living alone (no or yes), occupational status (employed, retired, or never employed), years of education (≥ 13, 10–12, or < 10 years), drinking status (none, past, or current), and smoking status (none, past, or current) were determined. The annual equivalent income was calculated by dividing the household income by the square root of the number of household members and categorized into three groups: ≥ $40,000; $20,000–$39,999; or < $20,000 per year (1 dollar = 100 yen). Disease status included hypertension, stroke, cardiovascular disease, diabetes mellitus, hyperlipidemia, musculoskeletal disorders, and cancer, which was assessed with yes or no answers. Body mass index was calculated from self-reported height and weight (kg/m^2^), with participants being categorized as either underweight (< 18.5), normal (18.5–24.9), or overweight/obese (≥ 25.0). Instrumental activities of daily living was assessed using the Tokyo Metropolitan Institute of Gerontology Index of Competence^24^ that examines five activities that people may perform in daily life, such as (1) using public transportation, (2) shopping for daily necessities, (3) preparing meals, (4) paying bills, and (5) handling their own banking. Each item was scored as 1 for yes (able to perform) or 0 for no; and participants were classified as good (5 points) or poor (≤ 4 points). The following potential mediators between watching sports and depressive symptoms were assessed: social cohesion (i.e., general trust, norms of reciprocity, and attachment to the neighborhood) and social network with friends (i.e., frequency at which participants met with friends and the number of friends met). General trust, norms of reciprocity, and attachment to the neighborhood were determine as yes (very and moderately) or no (neutral, slightly, and not at all)^25^. Frequency at which participants met with friends was categorized as follows: ≥ 4 times/week, 2–3 times/week, 1 time/week, 1–3 times/month, or a few times a year or less. The number of friends met was categorized as 0, 1–2, 3–5, 6–9, or ≥ 10.

### Statistical analysis

Multilevel mixed-effects Poisson regression analysis (the individual as level 1 and the municipality as level 2) with random intercepts and fixed slopes was performed to examine the association between watching sports and depressive symptoms while separately incorporating each mode of watching sports (i.e., on-site and via TV/Internet) into the regression models. When the incidence of an outcome of interest is common in the study population (> 10%), the adjusted odds ratio derived from the logistic regression can no longer approximate the risk ratio^26^. The prevalence of depressive symptoms in the present study was 21.4%; therefore, we applied Poisson regression and calculated the prevalence ratio (PR) and its 95% confidence interval (CI). The reference category for each mode was non-spectators. The next three models included the following: Model 1, frequency of watching sports, age groups, and sex; Model 2, Model 1 + potential confounders; and Model 3, Model 2 + potential mediators. Moreover, mediation analyses were conducted to calculate the proportion explained by the mediating effect of each social cohesion and network variable between watching sports and depressive symptoms based on the method described by Discacciati et al^27^. Apart from whole-sample analysis, subgroup analysis according to frequency of playing sports and exercise activities (≥ 1 or < 1 day/week) was conducted to confirm our hypothesis that depressive symptoms are less common among those watching sports frequently, regardless of whether they regularly play sports and exercise. Granted that older adults watching sports indeed had increased tendency to play sports, our subgroup analysis would help determine the relationship between watching sports and depressive symptoms more clearly than merely adjusting for playing sports. To address potential bias caused by missing values, we adopted multiple imputation under the missing at random assumption (i.e., a missing mechanism is related to the other variables measured in the same survey for that participant). Incomplete variables were imputed using a multivariate normal imputation method. We created 20 imputed datasets using all the variables introduced in the current analyses, after which estimated parameters were combined using Rubin’s combination methods^28,29^. Stata/MP 16.1 (StataCorp, College Station, Texas, USA) was used for all statistical analyses, with *P* < 0.05 indicating the statistical significance.

## Results

Table 1 summarizes the descriptive data according to sports and exercise status before performing multiple imputation for missing values. Among the 21,317 analyzed samples, 4559 (21.4%) had depressive symptoms. Moreover, among older adults who played sports/exercised at least once a week and less than once a week, 28.8% and 16.2% watched sports on-site once or more often a year, while 86.2% and 77.5% watched sports on TV/Internet, respectively.

**Table 1:** Characteristics of the participants by each playing sports/exercise activities status (n = 21,317).

	Playing sports/exercise activities					
	≥ 1 day/week (n = 12,418)		< 1 day/week (n = 7053)		Missing (n = 1846)	
	n	%	n	%	n	%
**Depressive symptoms**						
No (GDS < 5)	10,071	81.1	4971	70.5	1224	66.3
Yes (GDS ≥ 5)	2144	17.3	1945	27.6	470	25.5
Missing	203	1.6	137	1.9	152	8.2
**Playing sports/exercise activities**						
Zero			3448	48.9		
A few days/year			1374	19.5		
1–3 days/month			2231	31.6		
1 day/week	2178	17.5				
≥ 2 days/week	10,240	82.5				
Missing					1846	100.0
**Watching sports on-site**						
Zero	8480	68.3	5630	79.8	427	23.1
A few times/year	2974	24.0	1001	14.2	60	3.3
1–3 times/month	362	2.9	105	1.5	17	0.9
≥ 1 time/week	233	1.9	38	0.5	18	1.0
Missing	369	3.0	279	4.0	1324	71.7
**Watching sports via TV/Internet**						
Zero	1424	11.5	1364	19.3	158	8.6
A few times/year	2552	20.6	1724	24.4	126	6.8
1–3 times/month	3405	27.4	1711	24.3	117	6.3
≥ 1 time/week	4752	38.3	2030	28.8	159	8.6
Missing	285	2.3	224	3.2	1286	69.7
**Sex**						
Men	6166	49.7	3464	49.1	694	37.6
Women	6252	50.4	3589	50.9	1152	62.4
**Age groups (years)**						
65–69	2899	23.4	2162	30.7	293	15.9
70–74	3948	31.8	2035	28.9	415	22.5
75–79	3271	26.3	1441	20.4	457	24.8
80–84	1665	13.4	891	12.6	405	21.9
≥ 85	635	5.1	524	7.4	276	15.0
**Marital status**						
Married	9202	74.1	5093	72.2	1149	62.2
Unmarried	3076	24.8	1864	26.4	582	31.5
Missing	140	1.1	96	1.4	115	6.2
**Living alone**						
No	10,463	84.3	5970	84.6	1482	80.3
Yes	1836	14.8	1013	14.4	297	16.1
Missing	119	1.0	70	1.0	67	3.6
**Occupational status**						
Employed	3068	24.7	2674	37.9	235	12.7
Retired	8182	65.9	3562	50.5	471	25.5
Never employed	649	5.2	417	5.9	90	4.9
Missing	519	4.2	400	5.7	1050	56.9
**Education (years)**						
≥ 13	4298	34.6	1907	27.0	371	20.1
10–12	5379	43.3	3142	44.6	613	33.2
< 10	2454	19.8	1830	26.0	710	38.5
Missing	287	2.3	174	2.5	152	8.2
**Annual equivalent income**						
≥ $40,000	1504	12.1	838	11.9	117	6.3
$20,000–$39,999	4655	37.5	2441	34.6	340	18.4
< $20,000	4954	39.9	2960	42.0	795	43.1
Missing	1305	10.5	814	11.5	594	32.2
**Drinking status**						
None	5457	43.9	3229	45.8	932	50.5
Past	1236	10.0	725	10.3	177	9.6
Current	5320	42.8	2831	40.1	584	31.6
Missing	405	3.3	268	3.8	153	8.3
**Smoking status**						
None	7251	58.4	3928	55.7	1176	63.7
Past	3967	32.0	2037	28.9	413	22.4
Current	1030	8.3	954	13.5	167	9.1
Missing	170	1.4	134	1.9	90	4.9
**Disease status (multiple answer)**						
Hypertension (yes)	5224	42.1	3106	44.0	814	44.1
Stroke (yes)	283	2.3	148	2.1	48	2.6
Cardiovascular disease (yes)	1059	8.5	676	9.6	156	8.5
Diabetes mellitus (yes)	1635	13.2	929	13.2	230	12.5
Hyperlipidemia (yes)	2008	16.2	933	13.2	157	8.5
Musculoskeletal disorders (yes)	1175	9.5	701	9.9	199	10.8
Cancer (yes)	509	4.1	302	4.3	53	2.9
Missing	463	3.7	273	3.9	125	6.8
**Body mass index (kg/m**^**2**^**)**						
< 18.5	801	6.5	444	6.3	125	6.8
18.5–24.9	8756	70.5	4536	64.3	1111	60.2
≥ 25	2490	20.1	1812	25.7	395	21.4
Missing	371	3.0	261	3.7	215	11.7
**Instrumental activities of daily living**						
Good	11,423	92.0	6194	87.8	1553	84.1
Poor	706	5.7	629	8.9	138	7.5
Missing	289	2.3	230	3.3	155	8.4
**General trust**						
No	3107	25.0	2160	30.6	604	32.7
Yes	9090	73.2	4757	67.5	1121	60.7
Missing	221	1.8	136	1.9	121	6.6
**Norms of reciprocity**						
No	5198	41.9	3263	46.3	777	42.1
Yes	6961	56.1	3632	51.5	891	48.3
Missing	259	2.1	158	2.2	178	9.6
**Attachment to the neighborhood**						
No	2201	17.7	1568	22.2	419	22.7
Yes	10,021	80.7	5361	76.0	1296	70.2
Missing	196	1.6	124	1.8	131	7.1
**Frequency of meeting friends**						
A few times a year or less	2837	22.9	2331	33.1	504	27.3
1–3 times/month	2812	22.6	1891	26.8	406	22.0
1 time/week	1740	14.0	854	12.1	201	10.9
2–3 times/week	2747	22.1	1013	14.4	326	17.7
Almost every day	2074	16.7	839	11.9	220	11.9
Missing	208	1.7	125	1.8	189	10.2
**Number of friends met**						
0	786	6.3	812	11.5	157	8.5
1–2	1965	15.8	1573	22.3	348	18.9
3–5	2979	24.0	1718	24.4	470	25.5
6–9	1690	13.6	897	12.7	234	12.7
≥ 10	4780	38.5	1930	27.4	444	24.1
Missing	218	1.8	123	1.7	193	10.5

Note: Data are from the Japan Gerontological Evaluation Study (JAGES), a nationwide mail survey that was conducted from December 2019 to January 2020. Missing values were imputed by using a multivariate normal imputation method in the main regression analysis.

GDS: Geriatric Depression Scale.

Table 2 shows the results of multilevel mixed-effects Poisson regression analysis examining the relationship between watching sports on-site and depressive symptoms. According to whole-sample analysis, Model 1 showed that participants who watched sports on-site, regardless of frequency, had a lower prevalence of depressive symptoms than those who did not. Those who watched a few times/year (PR, 0.70; 95% CI, 0.65–0.74) or 1–3 times/month (PR, 0.66; 95% CI, 0.53–0.82) were less likely to develop depressive symptoms compared to non-spectators even after adjusting for potential confounders in Model 2. However, no significant relationship between watching sports and depressive symptoms was noted among those who watched once a week or more often (PR, 0.84; 95% CI, 0.66–1.06). In Model 3, which introduced social cohesion and networks as potential mediators, PRs changed by more than 0.1 and were close to 1.0 at each watching frequency. A subgroup analysis of those playing sports and exercise activities once a week or more often confirmed similar results. Another a subgroup analysis of those playing less frequently than once a week showed that those watching a few times/year had less frequent depressive symptoms compared to non-spectators after adjusting for potential confounders, although the overall association was weakened (PR, 0.81; 95% CI, 0.71–0.92).

**Table 2:** Prevalence ratios (PRs) and 95% confidence intervals (CIs) of depressive symptoms according to frequency of watching sports on-site.

	Model 1			Model 2			Model 3		
	PR	95% CI	*P*	PR	95% CI	*P*	PR	95% CI	*P*
**All (n = 21,317)**									
Zero	1.00			1.00			1.00		
A few times/year	0.60	(0.55–0.64)	< 0.001	0.70	(0.65–0.74)	< 0.001	0.80	(0.74–0.85)	< 0.001
1–3 times/month	0.54	(0.43–0.68)	< 0.001	0.66	(0.53–0.82)	0.001	0.79	(0.64–0.97)	0.026
≥ 1 time/week	0.72	(0.56–0.91)	0.007	0.84	(0.66–1.06)	0.148	1.02	(0.82–1.27)	0.873
Playing sports/exercise activities, ≥ 1 day/week (n = 13,372–13,483)*									
Zero	1.00			1.00			1.00		
A few times/year	0.59	(0.54–0.64)	< 0.001	0.64	(0.58–0.69)	< 0.001	0.75	(0.68–0.82)	< 0.001
1–3 times/month	0.55	(0.41–0.74)	< 0.001	0.61	(0.45–0.82)	0.001	0.74	(0.55–1.00)	0.047
≥ 1 time/week	0.79	(0.59–1.06)	0.118	0.83	(0.62–1.11)	0.201	1.00	(0.76–1.32)	0.982
Playing sports/exercise activities, < 1 day/week (n = 7834–7945)*									
Zero	1.00			1.00			1.00		
A few times/year	0.71	(0.63–0.81)	< 0.001	0.81	(0.71–0.92)	0.001	0.89	(0.78–1.01)	0.073
1–3 times/month	0.66	(0.45–0.97)	0.033	0.76	(0.53–1.09)	0.139	0.88	(0.62–1.23)	0.449
≥ 1 time/week	0.81	(0.47–1.41)	0.454	0.84	(0.52–1.36)	0.483	1.00	(0.64–1.56)	0.993

*Estimation sample varies across imputations.

Depressive symptoms were defined as a 15-item Geriatric Depression Scale score ≥ 5.

Model 1, adjusted for sex and age groups. Model 2, model 1 + frequency of playing sports/exercise, marital status, living alone, occupational status, years of education, equivalent income, drinking, smoking, disease status, body mass index, and instrumental activities of daily living. Model 3, model 2 + general trust, norms of reciprocity, attachment to the neighborhood, frequency of meeting friends, and number of friends met.

Table 3 shows the results of the multilevel mixed-effects Poisson regression analysis elucidating the relationship between watching sports via TV/Internet and depressive symptoms. Accordingly, whole-sample analysis showed that the prevalence of depressive symptoms was lower for participants who watched sports via TV/Internet than those who did not after adjusting for potential confounders in Model 2. Moreover, a dose–response relationship was confirmed (PRs were 0.86, 0.79, and 0.71 in those who watched a few times/year, 1–3 times/month, and ≥ 1 time/week, respectively). The introduction of potential mediators in Model 3 generally diminished the relationship (PRs were 0.92, 0.89, and 0.83, respectively). Similar results were confirmed in a subgroup analysis of those playing sports and exercise activities once a week or more often. Even a subgroup analysis of those playing less frequently than once a week showed that those watching 1–3 times/month (PR, 0.85; 95% CI, 0.77–0.95) or once a week or more often (PR, 0.75; 95% CI, 0.68–0.83) had less frequent depressive symptoms compared to non-spectators after adjusting for potential confounders.

**Table 3:** Prevalence ratios (PRs) and 95% confidence intervals (CIs) of depressive symptoms according to frequency of watching sports via TV/Internet.

	Model 1			Model 2			Model 3		
	PR	95% CI	*P*	PR	95% CI	*P*	PR	95% CI	*P*
**All (n = 21,317)**									
Zero	1.00			1.00			1.00		
A few times/year	0.76	(0.71–0.81)	< 0.001	0.86	(0.80–0.93)	< 0.001	0.92	(0.86–0.98)	0.016
1–3 times/month	0.65	(0.60–0.71)	< 0.001	0.79	(0.74–0.86)	< 0.001	0.89	(0.83–0.96)	0.001
≥ 1 time/week	0.56	(0.52–0.61)	< 0.001	0.71	(0.66–0.76)	< 0.001	0.83	(0.77–0.88)	< 0.001
Playing sports/exercise activities, ≥ 1 day/week (n = 13,372–13,483)*									
Zero	1.00			1.00			1.00		
A few times/year	0.72	(0.65–0.80)	< 0.001	0.79	(0.72–0.87)	< 0.001	0.86	(0.78–0.95)	0.002
1–3 times/month	0.64	(0.57–0.73)	< 0.001	0.73	(0.65–0.82)	< 0.001	0.84	(0.75–0.94)	0.002
≥ 1 time/week	0.57	(0.51–0.63)	< 0.001	0.66	(0.60–0.73)	< 0.001	0.79	(0.72–0.88)	< 0.001
Playing sports/exercise activities, < 1 day/week (n = 7834–7945)*									
Zero	1.00			1.00			1.00		
A few times/year	0.83	(0.75–0.91)	< 0.001	0.92	(0.84–1.02)	0.114	0.97	(0.89–1.07)	0.567
1–3 times/month	0.72	(0.65–0.80)	< 0.001	0.85	(0.77–0.95)	0.003	0.93	(0.84–1.03)	0.168
≥ 1 time/week	0.64	(0.58–0.70)	< 0.001	0.75	(0.68–0.83)	< 0.001	0.84	(0.77–0.92)	< 0.001

* Estimation sample varies across imputations.

Depressive symptoms were defined as a 15-item Geriatric Depression Scale score ≥ 5.

Model 1, adujusted for sex and age groups. Model 2, model 1 + frequency of playing sports/exercise, marital status, living alone, occupational status, years of education, equivalent income, drinking, smoking, disease status, body mass index, and instrumental activities of daily living. Model 3, model 2 + general trust, norms of reciprocity, attachment to the neighborhood, frequency of meeting friends, and number of friends met.

Supplementary Table 1 displays all PRs and 95% CIs in Model 3, while Supplementary Table 2 shows social cohesion and network according to frequency of watching sports. Older adults who watch sports both on-site and via TV/Internet had higher social cohesion and networks relative to non-spectators, while those who had rich social cohesion and network had a lower risk of depression. Supplementary Table 3 presents the results of the mediation analyses. All the social cohesion and network variables exerted a significant mediating effect between watching sports and depressive symptoms in the whole-sample analysis. The proportion explained by the mediating effect was 9.6%–23.7%.

## Discussion

To the best of our knowledge, this has been the first study to elucidate the relationship between watching sports on-site and via TV/Internet and depressive symptoms in older adults. Overall, the current study found that (1) the prevalence of depressive symptoms was lower among those who watched sports on-site at moderate frequency (i.e., several times a year to a month) and decreased as the frequency of watching sports via TV/Internet increased; (2) the relationship between watching sports and depressive symptoms was confirmed, regardless of whether they played sports or engaged in exercise activities; and (3) social cohesion and network exerted a mediating effect between watching sports and depressive symptoms.

The results presented herein support the findings of an intervention study that encouraged older adults to watch professional baseball games on-site^5^. In the aforementioned study, 29 older adults in the intervention group watched professional baseball games for a median of six times during the two-month intervention period, with the intervention group having significantly greater relief in depressive symptoms than the control group. The authors of the study inferred that depressive symptoms could be alleviated through the excitement and happiness induced by watching the performance of skilled professional athletes in a stadium environment that differs from everyday life^5^. The present study, on the other hand, evaluated not only watching professional sports at a stadium but also local sports activities. It is quite possible that watching and cheering for our favorite sports, teams, and players, including friends and family, could elicit enjoy and happiness despite the relatively low competitiveness. However, we note that those watching on-site once a week or more often did not have a low prevalence of depressive symptoms. Reports have shown that increased aggression and drinking intentions are some of the negative aspects exhibited by men watching sports^30^. The authors of that study also evaluated self-rated happiness among men before and after a rugby football game and found that winning did not increase happiness but drawing or losing decreased it^30^. We therefore infer that older adults who watch sports on-site every week might dwell on the results of the match or become obliged to be involved regardless of their own intentions rather than enjoying watching sports.

Watching sports via TV/Internet, which is quite easier than that on-site, was a common behavior observed even in older adults who played sports and exercised infrequently or not at all given that approximately 30% of the participants included herein watched sports once a week or more often. The dose–response relationship between watching sports and depression prevention might be a meaningful finding from the perspective of public health. In general, screen time, such as watching TV or computer, have been used as a proxy for physical inactivity and treated as a risk for obesity and non-communicable diseases^12,13^. Although viewing time of TV sports programs was not associated with physical activity among older adults, it was still reported to increase the risk of obesity^14^. Thus, while the negative aspects have been emphasized so far, different contexts regarding their association with depressive symptoms likely exist. According to a report investigating what kind of sports is being watched on TV in Japan, more than half of older adults watched the following: professional baseball (61.6%), Sumo (60.2%), games of the national soccer team (60.1%), marathon or Ekiden relay race (57.4%), figure skating (54.8%), and high school baseball (52.6%)^31^. Given the considerable popularity of these events in Japan, watching the players and teams playing competitively via TV/Internet can induce excitement and happiness and may have a positive effect on mental health.

We found that spectators tended to have richer social cohesion and networks with friends compared to non-spectators, which weakened the relationship between watching sports and depressive symptoms when added into the regression models. Furthermore, they exerted a significant mediating effect. These results suggest that social cohesion and networks may be one of the pathways by which watching sports reduces the risk of depressive symptoms. Reports have shown that older adults with rich social cohesion, evaluated using the same components employed herein (i.e., general trust, norms of reciprocity, and attachment to the neighborhood) had a lower risk of depression^32^. Thus, a fertile social networks with friends undoubtedly prevents depressive symptoms among older individuals^33,34^. The association between social cohesion and networks and depressive symptoms identified in Model 3 of the present study (see Supplementary Table 1) is consistent with those described in earlier studies. The promotion of health by connecting with the community and society through involvement in sports, in various manners apart from just playing, would definitely coincide with the concept of The Second Sport Basic Plan^6^ and Healthy Stadia^7,8^.

The strength of the present study was that it investigated the relationship between watching sports and depressive symptoms among older adults using a large, nationwide, and population-based sample not limited to specific types of sports, teams, or regions. However, some limitations of this study warrant discussion. First, given that reverse causality could occur due to the cross-sectional design, further longitudinal studies are required to address this limitation. Second, we could not consider the how long participants were familiar with watching sports or the time spent per session for each mode given the spatial constraints of the questionnaire, although information on frequency had been collected. Third, watching sports is a common and popular activity even in younger age groups; however, whether the results observed in this study can be generalized to those age groups is unclear. Future studies should collect information from various age groups on familiarity with watching sports or the time spent per session for each mode to determine the optimal frequency that would yield the greatest benefit.

## Conclusion

The current study found that older adults who watched sports on-site several times a year to a month have lower risk of depressive symptoms compared to those who did not. Our results also suggested that the more frequently they watched sports via TV or the Internet, the greater the association observed. These relationships are likely to be obtained regardless of whether they regularly played sports and exercised. Watching sports may directly and indirectly, via social cohesion and networking with friends, lower risk of depressive symptoms. Distributing sports game tickets or discount coupons to older adults or enhancing sports TV and Internet programs might be an effective population-based strategy for preventing depression. In addition, in a situation where our “doing/playing” sports activities is severely restricted owing to the COVID-19 pandemic, watching sports (particularly via TV and the Internet) may become more useful for preventing depression in the near future.

## Supplementary information

Supplementary Table 1.

Supplementary Table 2.

Supplementary Table 3.

## Data Availability

The data underlying this study is from the JAGES and contain sensitive information. Data for research purposes is available upon request. Requests for the JAGES data can be made to e-mail: dataadmin.ml@jages.net.
